# Script Concordance Test in Pharmacology: Maiden experience from a Medical School in India

**DOI:** 10.30476/jamp.2020.85163.1168

**Published:** 2020-07

**Authors:** MANDEEP KAUR, SHWETA SINGLA, RAJIV MAHAJAN

**Affiliations:** 1 Department of Pharmacology, Adesh Institute of Medical Sciences & Research, Bathinda, Punjab, India

**Keywords:** Medical education, Pharmacology, Medical school

## Abstract

**Introduction:**

Script concordance test (SCT) is an innovative tool to teach and assess the clinical reasoning skills of medical students. It is the key aspect of clinical competency that enables the medical graduates to progress from novice to practicing general practitioner. SCT was used the first time in pharmacology to inculcate clinic reasoning skills in medical students by focusing on the topic of pharmacotherapy.

**Methods:**

A SCT with a total of 18 questions, with 15 questions having 3 items each, one having four items and two questions having two items each was administered to 170 second year undergraduate medical students in the subject of pharmacology to assess the clinical reasoning skills. It was an interventional study conducted using convenience sampling technique with a sample size of 170. Aggregate scoring method was used to do the scoring obtained from the answers given by 10 expert-panel members in the field of pharmacology, which were used as an answer key to do the final scoring of the students. Descriptive statistics were computed using Students t test. SCT conduct included a small-group feedback session to the students post-SCT. A feedback questionnaire was administered to the students one week after the feedback session. Reliability of the SCT and feedback questionnaire was checked by calculating Cronbach alpha through Siegle reliability calculator. Content validity of the test as well as feedback questionnaire was done by the panel of experts included in the study.

**Results:**

Though the mean score of the students (27.68±4.59) was significantly lower than the expert panel (40.91±3.52), students were highly satisfied as they expressed that SCT enhanced their perceived clinical reasoning skills (median value=5) and critical thinking (median value=4). The Cronbach alpha for the test was 0.76. The students were also highly satisfied with the feedback given by the teachers after the SCT (median value=4).

**Conclusion:**

SCT enhances critical thinking and clinical reasoning skills of the students, as reported by them. With the conduct of feedback session post-SCT, it can be used as assessment for learning tool and can be well used in a para-clinical subject of pharmacology.

## Introduction

Good clinical reasoning skills are the utmost requirement in any clinical field. These skills are acquired by the students of medical college throughout their bachelor of medicine and bachelor of surgery (MBBS) course. Various assessment tools which are being used routinely to assess the learning of the students like multiple choice questions (MCQs), short answer questions, and oral viva questions are unable to probe the clinical reasoning skills of these medical students. To assess these skills, a special assessment tool- Script concordance Test (SCT) was developed and designed by Charlin et al. ( 1 ).

This test is based on the Script theory which asserts that whenever the clinicians or medical students are confronted with clinical situations, there is a triggering of Scripts ( 2 ). Script is an organizational knowledge used by the clinician to obtain the solution for the clinical problem ( 3 ). These scripts begin to appear when students encounter their first clinical case either by direct exposure to patient as in clinical subjects or in the form of a question based on clinical scenario as in basic or para-clinical subjects. Thereafter, these are further updated and refined throughout the MBBS course ( 4 ). 

Basically, SCT is based on the methodological theory of the organization of medical knowledge in the minds of medical students during their transition phase from novice to expert ( 5 ). In SCT, the examinees are given the case vignettes presenting a clinical situation which provides incomplete information to reach to a descision ( 6 ). It is followed by a series of questions (items) related to the diagnostic, investigative, or management problems. Each question is divided into three columns -first having initial hypothesis followed by the second column containing a new piece of clinical information that may or may not affect the initial hypothesis, followed by 5-point Likert scale to judge the effect of the new information on the initial hypothesis in the third column ( 4 ). The SCT test is unraveled by both students as well as the panel members expert in the field of respective discipline. Thereafter, the examinees’ responses were compared with those of the panel members.

SCT was successfully administered to the medical students in the clinical subjects of otolaryngology, psychiatry, emergency medicine, geriatric medicine, radiation oncology and dermatology ( 7 - 12 ). This test has been conducted in pharmacy as well as in basic clinical subjects but has not so far been conducted in pharmacology - a para-clinical subject during MBBS course ( 13 , 14 ). The objective of this study was to sensitize the faculty as well as students about the SCT. Another objective was to assess the student’s perception about performing the test in the para-clinical subject of pharmacology, with the help of feedback porforma.

## Methods

### *Construction and development of SCT questions*

SCT included 18 case vignettes with a total of 53 items in the test. Questions covered only the pharmacotherapeutics, the main applied aspect of pharmacology in patient care. Questions covered cardiovascular, blood and gastrointestinal portion of the pharmacology curriculum. This test comprised 18 questions with 3 items per question except for three questions in which one had four items and two had two items. Participants (students and expert panel) were asked to select the single best Likert response. A 5 point likert scale, ranging from -2 (Strongly contraindicated) to +2 (Strongly indicated) was used. An answer key was developed on the basis of the responses selected by members of the expert panels in the field of pharmacology. The final scoring of students as well as panel members was done. Sample SCT questions in Table 1 illustrate the structure and format of the SCT questions.

**Table 1:** Sample Script Concordance Test question

Q. A 25-year-old woman presented with complaints of headache, persistent rise in blood pressure (BP) from one week. Her present BP was 150/90 mm Hg. You made a diagnosis of essential hypertension		
If you were thinking of	Then you find out that	Then your plan of action becomes
1. Prescribing hydrochlorothiazide	Patient is on HMG Co-A reductase inhibitors for raised LDL	A B C D E
		-2 -1 0 +1 +2
2. Prescribing thiazide diuretic	Patient’s uric acid level are above normal range (8mg/dl)	A B C D E
		-2 -1 0 +1 +2
3. Prescribing calcium channel blocker	Patient’s urine pregnancy test (UPT) is +ve	A B C D E
		-2 -1 0 +1 +2

-2: Strongly contraindicated, -1: contraindicated, 0: more or less indicated, +1: indicated, +2: Strongly indicated

### *Subjects*

***Reference panel:*** Ten faculty members working in the department of pharmacology of medical colleges associated with tertiary care hospitals constituted the expert panel for the test. To facilitate the distribution of test and its receipt as well, was used online correspondence with the expert panel. 

***Medical students:*** A group of 170 students attending the classes of the 2nd year of MBBS in the subject of pharmacology participated in the study. Each student independently completed a paper-based version of the SCT. The students were sensitized about the concept of SCT one week before attempting the test. Feedback session for the students was held one week after SCT was conducted. After one week of feedback session, a feedback questionnaire was administered to the students. Only 166 questionnaires were returned. The questionnaire was evaluated for median values and satisfaction index.

For scoring, aggregate scoring method was used ( 15 ). According to it, there is no single best response to SCT items; several responses to each item may be acceptable. Initially, the responses of the expert panel members in the concerned field are scored (that is by treating each panelist as an examinee). Then, the credit is assigned to each response present on a Likert scale. This serves as a final answer key, which acts as a yardstick to do the scoring of the students ( 15 ). Credit is assigned to each response based on the number of expert panel members choosing that response. Credit of 1 point is given to the response that is chosen by the maximum number of panel members (modal answer). Other responses are attributed to a partial credit, proportionate to the number of experts choosing that response divided by the number of experts who have chosen the modal answer. Response not chosen by any panel member is awarded zero credit.

The purpose of having expert panel is to draw an answer key and the same answer key can be used to any number of students. For example, our reference panel comprised of 10 members who responded to the first item of the sample clinical scenario explained in Table 1, in the way shown in Table 2 as: none choose -2 response, one expert choose -1 response, two experts choose 0 response, five experts choose +1 response and two experts choose +2 response. Here, +1 response becomes the modal answer as it is answered by maximum number (5) of panel experts (Table 2). Thus, this answer is given credit of 1 (5/5; answer chosen by the number of experts divided by maximum number of experts who have chosen modal answer). Credit of zero is accorded to -2 response (0/5). -1 answer received credit of 0.2 (1/5), while 0 and +2 response received credit of 0.4, respectively (2/5).

**Table 2:** Example of the Script Concordance Test Scoring System

If you were thinking of	Then you find out that	Calculation	-2	-1	0	+1	+2
Prescribing hydrochlorothiazide	Patient is on HMG Co-A reducatse inhibitors for raised LDL	Response chosen by the panel members	0	1	2	5	2
		Score	0/5	1/5	2/5	5/5	2/5
		Credit per response	0	0.2	0.4	1	0.4

This exercise of expert panel scoring was conducted for every item and then scoring of all the items attempted by the candidates was conducted using expert panel’s answer key as exemplified in Table 2. Suppose, Roll No. 1 selects 0 response for the item shown in Table 2, he is awarded credit of 0.4, but if he selects -2 response, then he is given zero credit. 

Considering the items to be independent, scoring is done of each case (here 18 cases) by averaging the examinee’s scores over the number of items in that case rather than simply adding them. As our SCT included 18 cases with all having 3 items per case except 3 cases, where one case had 4 items and the other 2 cases had 2 items only. Averaging ensures that each case is not weighted by the number of questions it contains. At the end, the final score of each candidate was calculated by adding average scores of each case. Finally, the mean score was calculated.

### *Feedback and feedback questionnaire*

One week after the SCT, after ensuring all scoring, small group feedback session was conducted, with 25-30 students in each session. During these feedback sessions, the scripts were discussed, all possibilities were explored, and queries of the students were answered. One week post-feedback session, the feedback questionnaire was administered to the students. Content validity of the feedback questionnaire was ensured by giving it to the expert panel. Cronbach alpha was calculated for estimating the reliability. 166 students attended the feedback session and returned the complete questionnaire. The questionnaire had 14 questions related to SCT whose answers were to be given on 5-point Likert scale by the students (1= Strongly disagree, 2= Disagree, 3= Neither agree nor disagree, 4=Agree, 5=Strongly agree). The satisfaction index was calculated using the following formula ( 16 ).

$\mathrm{Satisfaction Index}=\frac{\left(n_{1}*1)+(n_{2}*2)+(n_{3}*3)+(n_{4}*4)+(n_{5}*5\right)}{\mathrm{N*5}}X100$

Where n_1_, n_2_, n_3_, n_4_, n_5_=number of students who marked the response 1, 2, 3, 4 and 5 on Likert Scale respectively; N=total number of students who participated 

Utility and feasibility of the SCT in pharmacology was established based upon informal feedback from the departmental faculty, expert panel members and from comparative scores of the students and expert panel.

Data was entered into Microsoft excel sheet. Descriptive statistics that included mean, standard deviation, minimum and maximum values and range of participants’ scores were computed. Student’s t-test was used to compare the mean scores of the experts and student.p < 0.05 was considered statistically significant. Reliability of the test and feedback questionnaire was estimated with Cronbach alpha coefficient using Siegle Reliability Calculator ( 17 ).

## Results

SCT which comprised of 18 case vignettes was administered to 170 second year students of MBBS and also to ten faculty members of reference panel who had 5-18 years of teaching experience in pharmacology. The reference panel had a mean score (40.91±3.52) significantly higher than that of the students (27.68±4.59) (p < 0.001; Table 3). The Cronbach alpha for the test was 0.76, while for feedback questionnaire it was 0.82.

**Table 3:** Comparison of the scores between groups

Group	Number	Mean ±S.D	Min	Max	Range	95% CI		df	p
						Lower	Upper		
Reference panel	10	40.91±3.52	37.02	49.16	12	10.31	16.15	178	<0.0001*
Students	170	27.68±4.59	16.13	42.2	26.07				

* p value=extremely statistically significant

**Figure 1:**
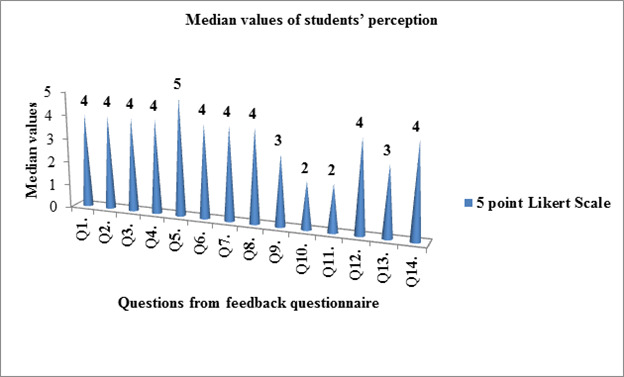
Median values of the students’ perception

A feedback questionnaire which comprised of 14 questions pertaining to the SCT was administered to 166 students who attended the feedback session one week after the test. It was further evaluated for satisfaction index and median values. Satisfaction index is an analytical tool designed for measuring the satisfaction of students with the type of the test administered to them. It was calculated in terms of percentages with the help of formula given above. Satisfaction index of the question regarding ‘enhancement of clinical reasoning skills by attempting SCT’ was the highest (90.12%), followed by lowest satisfaction index for question regarding ‘choice of oral examination over SCT’(48.07%) (Table 4). Median values for students’ perception was also calculated ( Figure1). It was observed that median value was the highest (=5; strongly agree) for the question regarding enhancing of clinical reasoning skills by SCT. The highest satisfaction index and median values for the question regarding enhancement of clinical reasoning skill was indicative of the fact that students are more interested in such type of tests that can facilitate their clinical reasoning ability and also establish the utility of the SCT.

**Table 4:** Satisfaction index of the students’ perceptions

No.	Statement	Satisfaction Index (%age)
1.	I was satisfied with this method of test.	83.61
2	I was satisfied with the feedback given by the teachers.	88.55
3.	I found the examination adequately covered the course.	83.25
4	I think my concepts related to above units were more clear after the SCT test.	81.69
5	I think this method of evaluation would enhance my clinical reasoning skills.	90.12
6	I think this method of evaluation would enhance my critical thinking.	86.02
7	I think this tests depicts real life scenarios.	84.1
8	I think that the SCT is a way to prepare me for clinical practice.	87.83
9	I would have preferred a multiple-choice examination (MCQs) instead of SCT.	62.77
10	I would have preferred routine theory class test instead of SCT.	52.17
11	I would have preferred an oral examination instead of SCT.	48.07
12	I think we should have more of such type of tests in the future.	79.64
13.	I found answering on likert scale difficult.	65.42
14.	I found answering on likert scale confusing.	70.36

Informal feedback from departmental faculty and expert panel members revealed that though framing case vignettes and items can be time consuming initially, they are quite feasible within the given resources, and no extra materialistic resources are required for their conduct.

## Discussion

As the competency-based medical education is gaining momentum across the globe, SCT is a step forward towards training the medical candidates and inculcating the clinical reasoning skills in the early stage of their development as a clinician. This study tested the feasibility and utility of SCT for the medical students as an evaluation tool for the first time in the subject of pharmacology. The test was easily constructed and written in a short period of time. Moreover, the panel members were also recruited easily, and the test was administered to them through online email platform. The scoring method was also not time-consuming, all factors pointing towards the feasibility of the SCT for medical students. 

The utility of the SCT in the medical students is to develop higher order thinking skills which can inculcate good clinical reasoning skills and critical thinking skills in the MBBS undergraduate students. The utility of this test was reflected by the SCT scores of the students which showed how closely the respondent is able to interpret clinical data when compared with that of experienced panel members in a given knowledge domain.

SCT scoring has found an important place in the evaluation of the test. The scoring scheme of the SCT assumes that the modal answer selected by the maximum number of panel members reflects the optimal reasoning skill under uncertain but ambiguous clinical situation. Though the answers selected by other panel members are also considered clinically valuable and receives partial credit. Scores of examinees reflect the degree of concordance with expert panel. If the examinee selects the modal answer, scoring will be higher, which indicates that his/her reasoning skills for interpretation of clinical case is highly in concordance with that of the experts in that field ( 1 ). As expected the scores obtained by the panel members were higher than those of the medical students. This difference reflects that SCT can distinguish the participants on the basis of their clinical experience ( 18 - 20 ). These inflated mean scores of the panel members in comparison to students are in accordance with the studies done by Piovezan et al. ( 4 ) and Kazour et al. ( 8 ). In our study, the reliability of SCT was estimated by the Cronbach alpha coefficient (=0.76), though good reliability is indicated when the coefficient is ≥ 0.80 ( 2 ). This is in accordance with the study done by Mathieu et al. ( 2 ) who reported a Cronbach alpha coefficient of 0.82 where SCT included 18 case vignettes with 60 items (almost similar to our study with 18 vignettes and 53 items).

In our study, the feedback session post SCT was also conducted and evaluated for the satisfaction index and median values of the students’ perception. To the best of our knowledge, only three studies conducted the feedback of students involved in research regarding SCT. According to Bursztejn et al.’s ( 12 ) study which was conducted on family medicine residents, it was indicated that the test was well accepted by experts and students, but there was no detailed evaluation regarding feedback of SCT in the study. In another study done by Duggan and Charlin et al. ( 19 ) on 5th year medical students, six questions regarding experience of using SCT was administered to the students and the data was expressed as percentage of respondents in each category for each variable. In another study by Mathieu et al. ( 2 ) on 5th year medical students of rheumatology, a questionnaire comprising of 9 questions for SCT assessment was administered to the students.The study noted that students agreed to participate in other SCT though they found the test uneasy and unfamiliar. None of the above three studies demonstrated the satisfaction index and median values of the students’ perception as it was done in our study. 

### *Limitations*

There were a few limitations in this study which must be taken into consideration. Our reference panel constituted 10 members expert in the field of pharmacology where some studies have used 15 member expert panel. Another limitations of the study is that by collecting feedback of the students via a survey about the utility of SCT, one can be assured about the perceived utility only; if in reality the SCT enhances the clinical reasoning skills can be gauzed by actually observing such trained students over a period of time in pragmatic conditions. Also, the test was conducted only at a single centre and in one specialty, so the results cannot be generalized to other medical students, and that require multicentric studies.

## Conclusion

SCT is a unique method to assess the clinical reasoning skills of medical students. It can be considered as a valid alternative to classical method of evaluation. The medical students were highly satisfied with this method of test as perceived from the satisfaction index and median values obtained for the questionnaire pertaining to the SCT. It can be successfully incorporated in regular curriculum of pharmacology.

## References

[1] Charlin B, Roy L, Brailovsky C, Goulet F, van der Vleuten C ( 2000). The script concordance test: a tool to assess the reflective clinician. Teach Learn Med.

[2] Mathieu S, Couderc M, Glace B, Tournadre A, Malochet-Guinamand S, Pereira B ( 2013). Construction and utilization of a script concordance test as an assessment tool for dcem3 (5th year) medical students in rheumatology. BMC Medical Education.

[3] Amini M, Shahabi A, Moghadami A, Rostamipour H, Kojuri J, Dehbozorgian M ( 2017). Psychpmetric characteristics of Script concordance test (SCT and its correlation with routine multiple choice (MCQ) in internal medicine department. Bio Med Res.

[4] Piovezan R, Custódio O, Cendoroglo M, Batista N, Lubarsky S, Charlin B ( 2012). Assessment of Undergraduate Clinical Reasoning in Geriatric Medicine: Application of a Script Concordance Test. Journal of the American Geriatrics Society.

[5] Charlin B, Gagnon R, Lubarsky S, Lambert C, Meterissian S, Chalk C ( 2010). Assessment in the Context of Uncertainty Using the Script Concordance Test: More Meaning for Scores. Teaching and Learning in Medicine.

[6] Dory V, Gagnon R, Vanpee D, Charlin B ( 2012). How to construct and implement script concordance tests: insights from a systematic review. Med Educ.

[7] Kania RE, Verillaud B, Tran H, Gagnon R, Kazitani D, Huy PTB ( 2011). Online script concordance test fro clinical reasoning assessment in otorhinolaryngology the association between performance an dclinical experience. Arch Otolaryngol Head Neck Surg.

[8] Kazour F, Richa S, Zoghbi M, El-Hage W, Haddad F ( 2016). Using the Script Concordance Test to Evaluate Clinical Reasoning Skills in Psychiatry. Academic Psychiatry.

[9] Boulouffe C, Charlin B, Vanpee D ( 2010). Evaluation of Clinical Reasoning in Basic Emergencies Using a Script Concordance Test. American Journal of Pharmaceutical Education.

[10] Piovezan R, Custódio O, Cendoroglo M, Batista N, Lubarsky S, Charlin B ( 2012). Assessment of Undergraduate Clinical Reasoning in Geriatric Medicine: Application of a Script Concordance Test. Journal of the American Geriatrics Society.

[11] Brazeau-Lamontagne L, Charlin B, Gagnon R, Samson L, van der Vleuten C ( 2004). Measurement of perception and interpretation skills during radiology training: utility of the script concordance approach. Med Teach.

[12] Bursztejn A, Cuny J, Adam J, Sido L, Schmutz J, de Korwin J ( 2011). Usefulness of the script concordance test in dermatology. Journal of the European Academy of Dermatology and Venereology.

[13] Funk K, Kolar C, Schweiss S, Tingen J, Janke K ( 2017). Experience with the script concordance test to develop clinical reasoning skills in pharmacy students. Currents in Pharmacy Teaching and Learning.

[14] Humbert A, Johnson M, Miech E, Friedberg F, Grackin J, Seidman P ( 2011). Assessment of clinical reasoning: A Script Concordance test designed for pre-clinical medical students. Med Teach.

[15] Norcini J, Shea J, Day S ( 1990). The use of the aggregate scoring for a recertification examination. Evaluation and the Health Professions.

[16] Tallapragada M Reply - How can I calculate patient satisfaction index according to NABH guideline? [Internet]. 2020. [Updated: 2 May 2020]. https://www.quora.com/How-can-I-calculate-patient-satisfaction-index-according-to-NABH-guideline.

[17] Siegle-Reliability-Calculator (version 1) - Cronbach's Alpha Split-Half(odd-even Correlation Spearman-Brown Prophecy Mean for Test Standard Deviation [Internet]. Coursehero.com. 2020 [cited 23 April 2020]. https://www.coursehero.com/file/16006365/Siegle-Reliability-Calculator-version-1/.

[18] Wan S ( 2015). Using the script concordance test to assess clinical reasoning skills in undergraduate and postgraduate medicine. Hong Kong Med J.

[19] Duggan P, Charlin B ( 2012). Summative assessment of 5thyear medical students’ clinical reasoning by script concordance test: requirements and challenges. BMC Med Edu.

[20] Gagnon R, Charlin B, Coletti M, Sauve E, van der Vleuten C ( 2005). Assessment in the context of uncertainty: how many members are needed on the panel of reference of a script concordance test?. Med Educ.

